# Impact of three-month tuberculosis preventive treatment (3HP) on IVF-ET outcomes in infertile women with tuberculosis infection: a retrospective before–after study

**DOI:** 10.1186/s12884-026-08725-x

**Published:** 2026-02-06

**Authors:** Jing Li, Qiuli Wu, Weixi Chen, Yanfang Wang, Shiming Xie, Tingting Li, Huisi Mai, Xiaoyan Liang

**Affiliations:** 1 Reproductive Medicine Center, The Sixth Affiliated Hospital, Sun Yat-sen University, Guangzhou, China; 2 Biomedical Innovation Center, The Sixth Affiliated Hospital, Sun Yat-sen University, Guangzhou, China

**Keywords:** Tuberculosis infection (TBI), Tuberculosis preventive treatment (TPT), 3 months of weekly rifapentine plus isoniazid (3HP), In vitro fertilization and embryo transfer (IVF-ET), Pregnancy outcomes, Recurrent implantation failure (RIF)

## Abstract

**Background:**

This retrospective before–after study aimed to evaluate whether tuberculosis preventive treatment (TPT) for tuberculosis infection (TBI, previously referred to as “latent TB infection” or LTBI) is associated with improved pregnancy outcomes in infertile women undergoing in vitro fertilization and embryo transfer (IVF-ET), and whether outcomes differ by the TPT duration and embryo origin.

**Methods:**

All participants initiated the WHO-recommended 3HP regimen (once-weekly isoniazid plus rifapentine; 12 doses over 3 months) after active TB was excluded. In this retrospective real-world cohort, the total documented duration of combination therapy varied in the medical record (3–18 months). Using a within-patient design, we compared pregnancy outcomes between embryo transfer cycles conducted before and after TPT. Post-TPT cycles were further stratified by TPT duration (3 months, 6 months, 12 months, 18 months) and embryo origin (embryos were cryopreserved from a cycle prior to TPT or derived after TPT ) to compare pregnancy outcomes within each stratum.

**Results:**

After TPT, biochemical pregnancy, clinical pregnancy, and live birth rates increased significantly, while early miscarriage rates decreased. Live birth rates did not differ significantly across the TPT duration subgroups (range: 32.69–39.29%). Among women with recurrent implantation failure, the live birth rate increased to 34.38% after TPT. Pregnancy outcomes did not differ by embryo origin.

**Conclusions:**

In infertile women with TBI undergoing IVF-ET, initiation of TPT with the guideline-recommended 3-month 3HP regimen was associated with improved pregnancy outcomes. In exploratory analyses, longer documented durations beyond 3 months were not associated with higher live-birth rates, supporting consideration of the standard course while acknowledging residual confounding inherent to this retrospective design.

**Supplementary Information:**

The online version contains supplementary material available at 10.1186/s12884-026-08725-x.

## Introduction

Tuberculosis (TB) remains a major global health threat. According to the World Health Organization (WHO) 2025 report, an estimated 10.7 million people fell ill with TB and 1.23 million died from the disease in 2024 [1]. While *Mycobacterium tuberculosis* most commonly causes pulmonary disease, its dissemination and manifestation as female genital tuberculosis (FGTB) holds particular relevance for reproductive health, representing a significant form of extrapulmonary TB [2, 3].

A substantial proportion of exposed individuals develop tuberculosis infection (TBI, formerly latent TB infection, LTBI), a state of persistent immune response to *M. tuberculosis* antigens without clinically active TB [4]. Although only 5–10% of individuals with untreated TBI progress to active TB [4], its impact on reproductive function is considerable. Evidence indicates that both FGTB and subclinical TBI can compromise fertility via multiple pathways: tubal obstruction and adhesions that hinder fertilization [5], ovarian involvement potentially diminishing reserve and oocyte quality [6–8], and endometrial dysfunction, characterized by compromised receptivity, involving altered molecular expression and impaired blood flow, which collectively contribute to implantation failure [8–10]. These mechanisms underlie the high rates of infertility associated with FGTB, particularly in endemic regions and among patients with delayed diagnosis. Furthermore, in pregnancies achieved via assisted reproduction, genital TB/TBI is associated with an increased risk of adverse outcomes, including maternal disseminated TB, preterm birth, and congenital tuberculosis in the infant [11–13]. Collectively, this dual burden of impaired fertility and increased obstetric risk provides a compelling rationale for administering tuberculosis preventive treatment (TPT) prior to assisted reproduction, aiming to both improve the likelihood of successful implantation and reduce associated maternal-fetal risks.

Although several TPT regimens are recommended by WHO and national guidelines (including the 3-month once-weekly isoniazid plus rifapentine regimen, 3HP), specific guidance for infertility patients remains limited [4]. This population must balance TPT timing, potential drug toxicity, and the scheduling of assisted reproductive technology (ART) cycles.

Therefore, this retrospective study aimed to evaluate, using a within-patient pre/post comparison design, whether completion of TPT is associated with improved key IVF-ET outcomes, including increased rates of clinical pregnancy and live birth, and a reduced rate of early miscarriage. We further explored whether outcomes varied by TPT duration or embryo origin (Transplanted embryos cryopreserved prior to TPT or derived after TPT). Our findings are intended to inform the development of clinical management pathways for infertile patients with TBI and to provide a reference for future clinical guidelines regarding pre-IVF screening, TPT strategy and timing, and subsequent embryo transfer protocols.

## Materials and methods

### Study Design, population and eligibility criteria

A retrospective, within-patient design was chosen as the most feasible and methodologically appropriate approach to (1) efficiently capture the real-world variation in TPT practice within this guideline-void clinical setting, and (2) inherently control for fixed, non-time-varying patient confounders (e.g., genetic factors, primary infertility etiology) by using each woman as her own control. Within this framework, the study population comprised infertile women (≤ 40 years old) undergoing IVF-ET at the Department of Reproductive Medicine of the Sixth Affiliated Hospital of Sun Yat-sen University (January 2017 – November 2023). Eligibility required evidence of TBI, specifically a tuberculin skin test (TST) induration ≥ 15 mm in an asymptomatic patient with normal chest imaging and no confirmatory evidence of active disease on laparoscopy, hysteroscopy, and/or endometrial biopsy when clinically indicated. Exclusion criteria were active TB, prior TB treatment, cancer, or immunosuppression, as these could confound both TB status and reproductive outcomes.

Diagnostic Approach and Rationale: The TST was utilized as the primary screening tool for TBI was based on the following considerations. First, it aligns with WHO guidelines, which state that either TST or an interferon-γ release assay (IGRA) is acceptable for testing [4]. Second, TST was the most readily accessible and the most economically feasible test in our clinical setting. Additionally, available T-SPOT.TB (an IGRA) results for a subset of TST-positive participants were analyzed, as detailed in Results section.

Rationale for the TST threshold: A TST induration threshold of ≥ 15 mm was selected to improve test specificity in our infertility population, among whom prior bacille Calmette-Guérin (BCG) vaccination is common or exposure to non-tuberculous mycobacteria and may cause false-positive results at lower thresholds, thereby enhancing the positive predictive value of the diagnosis.

### Treatment groups and study analysis

A total of 93 eligible women were included in this within-patient (self-controlled) analysis, contributing 317 embryo transfer (ET) cycles: 162 pre-TPT cycles performed before initiating TPT (pre-TPT group) and 155 cycles performed after completing TPT (post-TPT group). Thus, each woman served as her own control and pregnancy outcomes were compared between pre- and post-TPT cycles. While the within-patient design inherently controls for non-time-varying confounders (e.g., genetic factors), temporal biases are an unavoidable limitation.

For secondary analyses, post-TPT cycles were retrospectively stratified along two dimensions. First, by the observed total TPT duration (extracted from medical records) into four groups: 3, 6, 12, and 18 months, reflecting real-world practice variation rather than prospective assignment. Second, by embryo origin, operationally defined as whether embryos were cryopreserved from a cycle prior to TPT or derived from a cycle initiated after TPT completion. The study protocol was approved by the Ethics Committee of the Sixth Affiliated Hospital of Sun Yat-sen University (approval number: 2024ZSLYEC-290; Date of Approval: 6 June 2024 ).

### TPT regimen and monitoring

All participants were initiated on the WHO recommended 3HP regimen for the intended course of 3 months (12 doses). The regimen consisted of once-weekly isoniazid (INH) (based on the patient’s weight, 15 mg/kg, rounded up to the nearest 100 mg, maximum 900 mg) plus rifapentine (RPT) (750 mg for body weight 32.1–49.9 kg or 900 mg for ≥ 50.0 kg). Liver function was monitored at baseline and during treatment; medication was adjusted or interrupted if transaminase exceeded 3 times the upper limit of normal with symptoms, or 5 times without symptoms, based on clinician judgment.

Medical records showed heterogeneity in real‑world practice regarding how long clinicians continued preventive therapy beyond the intended 3‑month 3HP course. Specifically, while all women were initiated on the guideline‑based 3HP regimen (12 once‑weekly doses), a subset had documentation of continued once‑weekly isoniazid–rifapentine beyond 12 doses under physician supervision. We therefore categorized patients by the total documented duration of INH–RPT combination therapy (3, 6, 12, and 18 months) for exploratory subgroup analyses. We acknowledge that these extended durations represent off‑guideline practice and were not protocol‑assigned.

### TST protocol

Following the standardized Mantoux technique, 0.1 mL of a solution containing 5 tuberculin units of purified protein derivative (PPD; Beijing Sanroad Biological Products Co., Ltd., China) was injected intradermally into the volar forearm. The induration was measured 48–72 h post-injection, and the mean diameter (in mm) was calculated as the average of the horizontal and perpendicular readings.

### IVF-ET protocol

After completing TPT, patients proceeded either to frozen embryo transfer (using previously cryopreserved embryos)or to a new IVF cycle. For fresh cycles, controlled ovarian hyperstimulation was individualized (e.g., GnRH agonist/antagonist, or progestin-primed ovarian stimulation (PPOS) ). Subsequent oocyte retrieval, fertilization, embryo culture, and ultrasound-guided embryo transfer at the cleavage or blastocyst stage followed standardized protocols. Luteal phase support commenced on the day of oocyte retrieval or endometrial transformation, using vaginal progesterone gel, intramuscular progesterone, or oral dydrogesterone, alone or in combination.

### Pregnancy outcome definition

The primary outcome was live birth, defined as the delivery of a fetus with signs of life after 28 completed weeks of gestation. Secondary pregnancy outcomes included biochemical pregnancy (serum hCG ≥ 50 mIU/mL), clinical pregnancy (intrauterine gestational sac on ultrasound), early miscarriage (spontaneous pregnancy loss before 12 completed weeks) and ectopic pregnancy.

### Statistical analysis

Continuous variables were presented as means and standard deviations, and categorical variables as counts and proportions. Independent t-tests, one-way ANOVA, or nonparametric tests were used to compare continuous variables between groups, as appropriate. Chi-square or Fisher’s exact tests were employed for categorical variables. For the primary outcome of live birth, the effect of treatment is specifically reported as a Risk Ratio (RR) and an absolute Risk Difference (RD), each accompanied by its 95% Confidence Interval (CI). Multivariable logistic regression analysis was performed to identify factors predictive of live birth in the Post-TPT cycles including age, infertility duration, endometrial thickness, embryo stage at transfer, embryo origin and TPT duration. Adjusted odds ratios (aOR) with 95% CI and *P*-value were reported. All analyses were conducted using SPSS version 25.0, with a *P* value < 0.05 considered statistically significant. Analyses were performed primarily at the cycle level. As women could contribute multiple cycles, potential within-patient correlation that may inflate precision is acknowledged in the interpretation and listed as a study limitation.

Sample size consideration: As an exploratory retrospective study, no a priori sample size or power calculation was performed, as the sample comprised all eligible patients identified during the study period—a common approach of such designs. A post-hoc power analysis was not conducted, as it is mathematically determined by the observed *P* value and, being a well-recognized methodological limitation, provides no meaningful insight into the study’s true power.

## Results

### Tests for TB infection

Of the 93 enrolled TST-positive ( induration ≥ 15 mm) participants, 68 underwent dual testing with the T-SPOT.TB assay. Of these, 34 participants (50%) had a concordant positive result, while the other 34 (50.0%) had a discordant TST-positive/IGRA-negative result.

### Comparison of pregnancy outcomes between pre-TPT and post-TPT Cycles

A unadjusted paired comparison of pregnancy outcomes between cycles was performed before (pre-TPT, *n* = 162 ET cycles) the administration of TPT and after (post-TPT, *n* = 155 ET cycles) within the same women (Table 1). Post-TPT cycles exhibited significantly higher rates of biochemical pregnancy, clinical pregnancy and live birth compared with pre-TPT cycles (All *P* < 0.001). The live birth rate increased from 1.23% (2/162) to 36.13% (56/155) (RR = 29.30, 95% CI: 7.29–117.50; RD = 0.35, 95% CI: 0.28–0.42; *P* < 0.001). Moreover, the early miscarriage rate was substantially lower after TPT (13.43% vs. 62.50%, *P* < 0.001). No ectopic pregnancies occurred in the post-TPT cycles, whereas two ectopic pregnancies occurred in the pre-TPT cycles (*P* = 0.035). However, the time-related biases and within-subject correlation are unavoidable limitations in this analysis.

A subgroup analysis was conducted for 16 women with recurrent implantation failure (RIF), defined as the absence of implantation in two consecutive IVF/ICSI cycles where the cumulative number of transferred embryos was no less than four cleavage-stage embryos or no less than two blastocysts. Prior to TPT, the biochemical pregnancy rate was 10.42% (5/48 cycles) with no clinical pregnancies. After TPT, the biochemical pregnancy rate increased to 56.25% (18/32 cycles, *P* < 0.001), with a clinical pregnancy rate of 46.88% (15/32 cycles) and a live birth rate of 34.38% (11/32 cycles). Five of the 16 RIF women achieved a live birth in their first post-TPT cycle. Given the small sample size, these results should be interpreted as exploratory and hypothesis-generating.

**Table 1:** Pregnancy outcomes before (pre-TPT) and after TPT (post-TPT)

cycles, *n*	pre-TPT group	post-TPT group	*P*-value
	162	155	
Biochemical pregnancy rate, % (n)	20.99 (34/162)	46.45 (72/155)	<0.001
Clinical pregnancy rate, % (n)	9.88 (16/162)	43.23 (67/155)	<0.001
Ectopic pregnancy, % of clinical pregnancies (n/N)	12.50(2/16)	0(0/67)	0.035
Early miscarriage (< 12 weeks), % of clinical pregnancies (n/N)	62.50 (10/16)	13.43 (9/67)	<0.001
Live birth rate, % (n)	1.23(2/162)	36.13(56/155)	<0.001
Live birth rate, RR (95% CI)	1 (Reference)	29.30 ( 7.29–117.50)	<0.001^†^
Live birth rate, RD (95% CI)	— (Reference)	0.35 ( 0.28–0.42)	<0.001^†^

Comparisons between pre- and post-TPT cycles were unadjusted for covariates. ^†^ Represents the *P*-value for the live birth rate comparison applies to both the RR and RD estimates

### Comparison of pregnancy outcomes stratified by observed TPT duration (real-world practice variation)

A unadjusted paired comparison of pregnancy outcomes before and after TPT was performed within each TPT duration subgroup (3, 6, 12, and 18 months). Live birth rates were significantly higher after TPT in all subgroups (all *P* < 0.005; Supplementary Table 1). The absolute risk differences were substantial, ranging from 0.327 to 0.372 (all 95% CIs excluded zero).

Patient characteristics and outcomes after TPT across these subgroups are shown in Table 2. Except for a longer duration of infertility in the 12- and 18-month subgroups (*P* = 0.007), no significant differences were found in age, BMI, endometrial thickness, or the number of embryos transferred. Live birth rates after TPT were similar across the 3‑, 6‑, 12‑, and 18‑month subgroups (*P* = 0.933). In comparisons using the 3-month subgroup as reference, the risk ratios (and 95% CIs) were 1.06 (0.57–1.96) for 6 months, 1.01 (0.55–1.85) for 12 months, and 0.88 (0.52–1.49) for 18 months, all consistent with no significant difference.

**Table 2:** Post-TPT outcomes stratified by observed preventive-therapy duration (real-world practice variation)

cycles, *n*	3 months	6 months	12 months	18 months	*P*-value
	43	28	32	52	
Age, years	33.12 ± 3.41	32.64 ± 4.64	34.23 ± 3.58	34.12 ± 3.27	0.336
Duration of infertility, years	4.19 ± 3.53^a^	4.25 ± 2.26^a^	6.00 ± 2.69^b^	5.98 ± 3.41^b^	0.007
BMI, kg/m^2^	21.55 ± 2.22	21.71 ± 3.00	21.16 ± 2.45	20.60 ± 1.66	0.111
Endometrial thickness, mm	10.13 ± 2.33	10.34 ± 2.68	10.24 ± 1.81	9.80 ± 1.78	0.667
Number of embryo transferred, % (n)					0.900
1	39.53(17/43)	39.29(11/28)	40.63(13/32)	46.15(24/52)	
2	60.47(26/43)	60.71(17/28)	59.37(19/32)	53.85(28/52)	
Development stage of embryo transferred, % (n)					0.203
Cleavage stage	44.19(19/43)	42.86(12/28)	34.37(11/32)	25.00(13/52)	
Blastocyst	55.81(24/43)	57.14(16/28)	65.63(21/32)	75.00(39/52)	
Biochemical pregnancy rate, %(n)	46.51(20/43)	39.29(11/28)	50.00(16/32)	48.08(25/52)	0.851
Clinical pregnancy rate, % (n)	41.86(18/43)	39.29(11/28)	50.00(16/32)	42.31(22/52)	0.843
Early miscarriage (< 12 weeks), % of clinical pregnancies (n/N)	5.56(1/18)	0	25.00(4/16)	18.18(4/22)	0.177
Live birth rate, % (n)	37.21(16/43)	39.29(11/28)	37.50(12/32)	32.69(17/52)	0.933
Live birth rate, RR (95% CI)	1 (Reference)	1.06(0.57–1.96)	1.01 (0.55–1.85)	0.88 (0.52–1.49)	0.933^†^
Live birth rate, RD (95% CI)	—(Reference)	0.02(-0.19–0.23)	0.003(-0.19–0.20)	-0.45(-0.23–0.14)	0.933^†^

Different letters represent statistical significance between the groups

^†^ Represents the *P*-value for the live birth rate comparison applies to both the RR and RD estimates

Given the observational design and limited subgroup sizes, our data do not support a conclusion of formal equivalence or non‑inferiority across durations. Rather, we did not observe higher live‑birth rates with longer documented preventive‑therapy duration compared with the standard 3‑month course in this cohort. 

### Comparison of pregnancy outcomes between subgroups stratified by embryo origin

Patient characteristics and cycle outcomes were compared between subgroups based on whether embryos transferred post-TPT were cryopreserved from a cycle prior to TPT or derived from a cycle initiated after TPT completion (Table 3). BMI, infertility duration and number of embryos transferred were comparable between the subgroups (*P* > 0.05). However, patients in “Embryos cryopreserved prior to TPT” subgroup were older (34.16 ± 3.75 vs. 32.85 ± 3.45, *P* = 0.028) and had a higher proportion of blastocyst transfers (72.41% vs. 54.41%, *P* = 0.020) with thinner endometria (9.40 ± 1.49 vs. 10.94 ± 2.46 mm, *P* < 0.001). Despite these differences, the live birth rate was similar between the “Embryos cryopreserved prior to TPT” and “Embryos derived after TPT” subgroups (35.63% vs. 36.76%, *P* = 0.884). The effect estimates supported the absence of a meaningful difference (comparisons using the Embryos cryopreserved prior to TPT subgroup as reference, RR = 1.03, 95% CI: 0.68–1.58; RD = 0.01, 95% CI :-0.12–0.15).

**Table 3:** Characteristics and pregnancy outcomes between subgroups differentiated by embryo origin

cycles, *n*	Embryos cryopreserved prior to TPT subgroup	Embryos derived after TPT subgroup	*P*-value
	87	68	
Number of oocyte retrieved	10.72 ± 5.14	10.78 ± 4.91	0.876
Number of good-quality embryo	3.06 ± 3.24	3.50 ± 3.11	0.318
Age, years	34.16 ± 3.75	32.85 ± 3.45	0.028
Duration of infertility, years	5.41 ± 3.23	4.87 ± 3.21	0.296
BMI, kg/m^2^	21.22 ± 2.12	21.13 ± 2.51	0.820
Endometrial thickness, mm	9.40 ± 1.49	10.94 ± 2.46	<0.001
Number of embryo transferred, % (n)			0.866
1	42.53(37/87)	41.18(28/68)	
2	57.47(50/87)	58.82(40/68)	
Development stage of embryo transferred, % (n)			0.020
Cleavage stage	27.59(24/87)	45.59(31/68)	
Blastocyst	72.41(63/87)	54.41(37/68)	
Biochemical pregnancy rate, % (n)	49.43 (43/87)	42.65 (29/68)	0.401
Clinical pregnancy rate, % (n)	44.83 (39/87)	41.18 (28/68)	0.649
Early miscarriage (< 12 weeks), % of clinical pregnancies (n/N)	15.38 (6/39)	10.71 (3/28)	0.104
Live birth rate, % (n)	35.63(31/87)	36.76(25/68)	0.884
Live birth rate, RR (95% CI)	1 (Reference)	1.03(0.68–1.58)	0.884^†^
Live birth rate, RD (95% CI)	—(Reference)	0.01(-0.12–0.15)	0.884^†^

^†^ Represents the *P*-value for the live birth rate comparison applies to both the RR and RD estimates

### Factors associated with live birth in the post-TPT Cycles

Multivariable logistic regression was performed to identify factors independently associated with live birth in post-TPT cycles, adjusting for age, infertility duration, endometrial thickness, embryo stage at transfer, embryo origin, and TPT duration (Table 4). After adjustment, two factors were identified as independent positive predictors of live birth: blastocyst transfer (vs. cleavage-stage transfer: aOR = 3.501; 95% CI: 1.484–8.260; *P* = 0.004) and greater endometrial thickness (per 1 mm increase: aOR = 1.247; 95% CI: 1.035–1.502; *P* = 0.020). Neither TPT duration (overall *P* = 0.736) nor embryo origin (aOR = 0.998; 95% CI: 0.443–2.247; *P* = 0.995) showed a significant association with live birth.

**Table 4:** Multivariable logistic regression analysis identifying factors associated with live birth

variables	Live birth	
	aOR(95% CI)	*P* value
Age	0.992(0.898–1.097)	0.882
Duration of infertility	1.039(0.926–1.166)	0.517
Endometrial thickness	1.247(1.035–1.502)	0.020
Blastocyst (vs. Cleavage)	3.501(1.484–8.26)	0.004
Embryo origin (Derived after TPT vs. Cryopreserved prior to TPT)	0.998(0.443–2.247)	0.995
TPT duration		0.736
6 months vs. 3months	1.109(0.391–3.146)	0.845
12 months vs. 3months	0.903(0.313–2.609)	0.851
18 months vs. 3months	0.645(0.25–1.661)	0.363

## Discussion

In this within-patient before–after analysis, completion of TPT was associated with significantly higher biochemical pregnancy, clinical pregnancy, live birth rates, as well as a lower early miscarriage rate in women with TBI undergoing IVF-ET.

An observation in our cohort was the 50% discordance between TST and IGRA testing, which was entirely in the TST-positive/IGRA-negative pattern—the most common form of discordance [14]. This finding aligns with a large multicenter prospective cohort study in China, which, using QuantiFERON-TB Gold (a different IGRA method), reported only moderate overall agreement between TST and IGRA in the general population (81.06%; kappa coefficient 0.485) [15]. This demonstrates that the TST-positive/IGRA-negative pattern is a widespread phenomenon in our epidemiological context and may well be attributed to prior BCG vaccination [14], suggesting that our TST-defined cohort may have included cases with false-positive results. Given this finding stems from a subset of participants and its precision is accordingly limited, it may reflect a relevant context of diagnostic heterogeneity. In this context, an increase in the live birth rate to 36.13% was observed following TPT. This corresponds to a substantial absolute risk difference of 0.35 (95% CI: 0.28–0.42). Although the imprecision in the relative risk estimate (wide confidence interval) may partly reflect the diagnostic heterogeneity noted above, the clear benefit may strengthen the clinical relevance of the finding. Our results corroborate prior evidence of improved outcomes after TPT in infertile women with TBI [6]– [7]. Thus, TPT prior to assisted reproduction may represent a beneficial adjunct clinical approach to improving pregnancy outcomes in this patient population, although the exact magnitude of the relative benefit requires confirmation in larger, prospective cohorts.

Building on this, we specifically evaluated the association between TPT duration and live birth. Our analysis offers preliminary evidence that live birth rates were comparable among infertile women receiving TPT for durations ranging from 3 to 18 months. We fully acknowledge that the guideline-concordant 3HP regimen is a fixed 12-dose (3-month) course. In our cohort, the variation in the recorded “TPT duration” (3–18 months) reflects real-world physician-level practice variation in a guideline-void infertility setting, rather than a prospectively assigned protocol. Specifically, during the study period, infertility clinicians held differing views regarding how long preventive therapy should be continued before proceeding with embryo transfer, given concerns about possible subclinical TB–related inflammation and the lack of infertility-specific consensus pathways. Therefore, some patients received therapy beyond the standard course as an off-label, exploratory extension, based on individualized clinician judgment and patient preference.

Importantly, our study does not advocate prolonged therapy. Instead, we aimed to document this real-world variation and examine whether longer observed durations were associated with additional reproductive benefit. In our analyses, pregnancy outcomes after TPT were comparable across duration subgroups, and we did not observe evidence of improved live birth rates with longer durations. These findings suggest that completing a short-course, guideline-concordant 3-month 3HP regimen may be sufficient for many infertility patients with LTBI preparing for ART, potentially minimizing delays to IVF/ET. Given the observational design and limited subgroup sizes, these findings should be interpreted as hypothesis-generating and do not support formal claims of equivalence or non-inferiority across durations. And it should not be extrapolated to the management of active TB disease or confirmed female genital TB, which require standard multidrug treatment.

Given the potential clinical implications, we further analyzed outcomes in the subgroup of patients with RIF. Although the sample size is limited and statistical power is constrained, our exploratory analysis showed that RIF patients achieved a live birth rate of 34.38% (11/32 cycles) after TPT, compared to 0% (0/48 cycles) before treatment. While these preliminary findings are suggestive and point to a potential therapeutic avenue for RIF patients, they remain to be validated in larger, prospective studies.

To explore the potential biological basis for the observed improvement in pregnancy outcomes following TPT, we consider the pathophysiological mechanisms that may explain these outcomes. Beyond tubal factors, TBI is clinically associated with diminished ovarian reserve and poorer oocyte retrieval outcomes [16]– [17]. Furthermore, endometrial receptivity is compromised, evidenced by the downregulation of key implantation molecules (e.g., ITGαVβ3, MECA79, CDH1) and LIF-STAT3 signaling, alongside reports of reduced endometrial thickness and impaired endometrial blood flow in dormant genital tuberculosis [7, 9].Additionally, a pro-inflammatory milieu characterized by elevated Th1-type cytokines such as IFN-γ and IL-2 is observed, which contributes to an environment hostile to embryo implantation [18–20]. we hypothesize that the observed clinical improvement is mechanistically linked to immunomodulation. Specifically, by attenuating the detrimental immune milieu induced by TBI, TPT may facilitate a restoration of endometrial receptivity, thereby enhancing implantation and pregnancy success rates.

The embryo-origin analysis shows that favorable pregnancy outcomes after TPT were observed with both embryos cryopreserved before TPT and those generated after TPT. This may provide practical flexibility for patients who already have cryopreserved embryos. Although our data do not directly address ovarian reserve, this finding suggests (but does not prove) that patients with poor ovarian reserve diagnosed with TBI could consider embryo cryopreservation prior to TPT initiation. However, such a strategy remains hypothetical and requires validation in future studies.

While published case reports raise concern that ART and pregnancy may increase TB reactivation risk in susceptible individuals with FGTB or TBI [21–24], our study provides preliminary evidence that systematic screening followed by TPT may mitigate this risk. In our cohort, no TB-associated adverse pregnancy outcomes—such as miliary TB during pregnancy or postpartum, or neonatal congenital TB—were observed following IVF-ET. Although the study was not powered to detect rare events, this safety finding aligns with the protective premise of preventive therapy in this clinical setting.

Regarding safety, no serious adverse drug events were documented. All 93 patients (100%) initiating TPT were monitored monthly throughout follow-up with clinical review and liver function tests, and no patients discontinued treatment due to adverse events. Nonetheless, clinicians should remain vigilant for known potential side effects, including hepatotoxicity, hypersensitivity reactions, and drug-drug interactions associated with regimens containing isoniazid and rifapentine [25].

This study has several limitations. First, its retrospective before–after design is susceptible to confounding and, importantly, cannot control for time-related biases such as changes in clinical practice, natural variation in endometrial receptivity across cycles, or regression to the mean. Second, the analysis was conducted largely at the cycle level without accounting for within‑subject correlation, as many women contributed multiple treatment cycles, which may introduce within-subject correlation. Third, reliance on TST for enrollment may have introduced selection bias, as shown by the 50% discordance with IGRA which suggests inclusion of TST-false-positive individuals. Fourth, the single‑center setting and the limited sample size may limit the generalizability of the findings. Finally, longer‑term maternal and neonatal outcomes were not comprehensively assessed.

Prospective, multicenter studies employing improved diagnostic strategies, appropriate statistical methods for repeated cycles, and extended follow‑up term are needed to validate these results and to optimize TBI screening and treatment pathways in infertile populations.

## Conclusion

In summary, this retrospective within‑patient study suggests that in infertile women with TBI, initiation of the guideline‑recommended 3‑month 3HP tuberculosis preventive treatment is associated with improved IVF outcomes. In exploratory duration subgroup analyses, we did not observe evidence of incremental benefit with longer documented durations beyond the standard course; however, these comparisons are unadjusted and hypothesis‑generating. Prospective controlled studies are needed to confirm these findings, to better address time‑related confounding, and to define optimal screening and treatment pathways for this population.

## Supplementary Information

Supplementary Material 1.

## Data Availability

The datasets used and/or analysed during the current study are available from the corresponding author on reasonable request.

## Abbreviations

TB: Tuberculosis
TPT: Tuberculosis preventive treatment
TBI: Tuberculosis infection
LTBI: Latent TB infection
IVF-ET: In vitro fertilization and embryo transfer
TST: Tuberculin skin test
3HP: 3 months of weekly rifapentine plus isoniazid
RIF: Recurrent implantation failure
WHO: World health organization
FGTB: Female genital tuberculosis
ART: Assisted reproductive technology
IGRA: Interferon-γ release assay
ET: Embryo transfer
BCG: Bacille Calmette-Guérin

## References

[1] Global tuberculosis report 2025, World Health Organization. Geneva: ; 2025. Licence: CCBY-NC-SA3.0IGO. https://www.who.int/teams/global-programme-on-tuberculosis-and-lung-health/tb-reports/global-tuberculosis-report-2025.‘Accessed’ 2026/1/12.

[2] Vijay A, Tiwari N, Sharma A, Pandey G. Correlation of female genital tuberculosis and infertility: A comprehensive systematic Review, Meta-analysis, and female genital tuberculosis infertility pathway analysis. J Mid-Life Health. 2023;14(3):165–9. 10.4103/jmh.jmh_151_23 PMC10836443 38312757

[3] Tjahyadi D, Ropii B, Tjandraprawira KD et al. Female Genital Tuberculosis: Clinical Presentation, Current Diagnosis, and Treatment. Infectious Diseases in Obstetrics and Gynecology. 2022; 2022: 1–6. 10.1155/2022/3548190 PMC9699775 36438172

[4] WHO consolidated guidelines on tuberculosis. Module 1: prevention – tuberculosis preventive treatment, second edition. Geneva: World Health Organization. 2024. Licence: CC BY-NC-SA 3.0 IGO. 39298638

[5] Mondal R, Jaiswal N, Bhave P, Mandal P. Laparoscopic and hysteroscopic findings in women with sub-fertility and tuberculosis: A case series. Bjog-Int J Obstet Gy. 2024;131(7):929–40. 10.1111/1471-0528.17701 37973605

[6] Jirge PR, Chougule SM, Keni A, Kumar S, Modi D. Latent genital tuberculosis adversely affects the ovarian reserve in infertile women. Hum Reprod. 2018;33(7):1262–9. 29897442 10.1093/humrep/dey117

[7] Dam P, Shirazee HH, Goswami SK, et al. Role of latent genital tuberculosis in repeated IVF failure in the Indian clinical setting. Gynecol Obstet Inves. 2006;61(4):223–7. 10.1159/000091498 16479141

[8] Richa S, Anjali K, Sonal J, Akrati J. Analysis of the effect of female genital tuberculosis on ovarian reserve parameters. J Hum Reprod Sci. 2023;16(2):125–31. 37547096 10.4103/jhrs.jhrs_36_23 PMC10404021

[9] Subramani E, Madogwe E, Ray CD, et al. Dysregulated leukemia inhibitory factor and its receptor regulated signal transducers and activators of transcription 3 pathway: a possible cause for repeated implantation failure in women with dormant genital tuberculosis? Fertil Steril. 2016;105(4):1076–84. 26776907 10.1016/j.fertnstert.2015.12.015

[10] D’Attilio L, Santucci N, Bongiovanni B, Bay ML, Bottasso O. Tuberculosis, the disrupted Immune-Endocrine response and the potential thymic repercussion as a contributing factor to disease physiopathology. Front Endocrinol. 2018;9:214. 10.3389/fendo.2018.00214 PMC5938357 29765355

[11] Gai X, Chi H, Cao W, et al. Acute miliary tuberculosis in pregnancy after in vitro fertilization and embryo transfer: a report of seven cases. Bmc Infect Dis. 2021;21(1):913. 34488670 10.1186/s12879-021-06564-z PMC8419986

[12] Sands A, Santiago MT, Uduwana S, et al. Congenital tuberculosis after in vitro fertilization: A case for tuberculosis screening of women evaluated for infertility. Clin Infect Dis. 2023;76(3):e982–6. 35788281 10.1093/cid/ciac542

[13] Gai X, Chi H, Li R, Sun Y. Tuberculosis in infertility and in vitro fertilization-embryo transfer. Chin Med J-Peking 2024; 137(20). 10.1097/CM9.0000000000003255 PMC11479491 39169453

[14] Testing and Treatment of Latent Tuberculosis Infection in the United States. ‘Available at:’ https://npin.cdc.gov/publication/testing-and-treatment-latent-tuberculosis-infection-united-states

[15] Gao L, Lu W, Bai L, et al. Latent tuberculosis infection in rural china: baseline results of a population-based, multicentre, prospective cohort study. Lancet Infect Dis. 2015;15(3):310–9. 25681063 10.1016/S1473-3099(14)71085-0

[16] Yang X, Chen H, Kong X, et al. The effect of latent Mycobacterium tuberculosis infection on ovarian reserve and pregnancy outcomes among infertile women in China. Bmc Infect Dis; 2025. 10.1186/s12879-025-12424-x PMC12836983 41437221

[17] Wang Z, Zhang X, Dai B, Li D, Chen X. Analysis of the potential regulatory mechanisms of female and latent genital tuberculosis affecting ovarian reserve function using untargeted metabolomics. Sci Rep-Uk. 2024;14(1):9519. 10.1038/s41598-024-60167-7 PMC11045857 38664479

[18] Datta A, Chaudhuri AR, Chatterjee S, Chowdhury RG, Bhattacharya B. Role of endometrial cytokines of the female genital tract tuberculosis in the context of infertility. Blde Univ J Health Sci 2018; 3(1).

[19] Datta A, Bagchi B, Chatterjee S, et al. Role of TH-I based endometrial cytokines in latent genital tuberculosis and endometriosis causing infertility. SF J In Vitro Fertil Embryo Res. 2020;1(1):1001.

[20] Sharma JB, Sharma S, Sharma E, Dharmendra S, Singh S. Immune disturbances in female genital tuberculosis and latent genital tuberculosis. Am J Reprod Immunol. 2023;89(2):e13632. 36494901 10.1111/aji.13632

[21] Ma H, Sun J, Zhang L, et al. Disseminated hematogenous tuberculosis following in vitro Fertilization-Embryo transfer: A case report. Infect Drug Resist. 2021;14:4903–11. 34853518 10.2147/IDR.S332992 PMC8627859

[22] Cao W, Fu X, Li H, Bei J, Li L, Wang L. Tuberculosis in pregnancy and assisted reproductive technology. Drug Discoveries Ther. 2024;18(2):1007–2024. 10.5582/ddt.2024.01007 38631867

[23] Gull I, Peyser MR, Yaron Y, Jaffa AJ, Amit A, Lessing JB. The effect of an in-vitro fertilization pregnancy on a woman with genital tuberculosis. Hum Reprod. 1995;10(11):3052–4. 8747071 10.1093/oxfordjournals.humrep.a135846

[24] Li X, Qiu J, Ma Y, Cui T. June 30,. Reactivation of latent Mycobacterium tuberculosis infection with different outcomes after in vitro fertilization and embryo transfer: two case reports. Gynecology and Pelvic Medicine; Vol 6 (2023): Gynecology and Pelvic Medicine 2023.

[25] Latent tuberculosis infection. A guide for primary health care providers.

